# Structure and Dynamics of Mono- vs. Doubly Lipidated Rab5 in Membranes

**DOI:** 10.3390/ijms20194773

**Published:** 2019-09-26

**Authors:** Eileen Münzberg, Matthias Stein

**Affiliations:** Max Planck Institute for Dynamics of Complex Technical Systems, Molecular Simulations and Design Group, Sandtorstrasse 1, 39106 Magdeburg, Germany; edler@mpi-magdeburg.mpg.de

**Keywords:** GTPase, post-translation modification, molecular dynamics, lipid bilayer, diffusion

## Abstract

The Rab5 small GTPase is a regulator of endosomal trafficking and vesicle fusion. It possesses two adjacent cysteine residues for post-translational geranylgeranylation at its C-terminus for the protein to associate with the early endosome membrane. We compare the effect of mono-lipidification of only one cysteine residue with the doubly modified, fully functional Rab protein in both guanosine diphosphate (GDP)- and guanosine triphosphate (GTP)-bound states and in different membranes (one, three, and six-component membranes). Molecular simulations show that the mono-geranylgeranylated protein is less strongly associated with the membranes and diffuses faster than the doubly lipidated protein. The geranylgeranyl anchor membrane insertion depth is smaller and the protein–membrane distance distribution is broad and uncharacteristic for the membrane composition. The mono-geranylgeranylated protein reveals an unspecific association with the membrane and an orientation at the membrane that does not allow a nucleotide-specific recruitment of further effector proteins. This work shows that double-lipidification is critical for Rab5 to perform its physiological function and mono-geranylgeranylation renders it membrane-associated but non-functional.

## 1. Introduction

**Figure 1:**
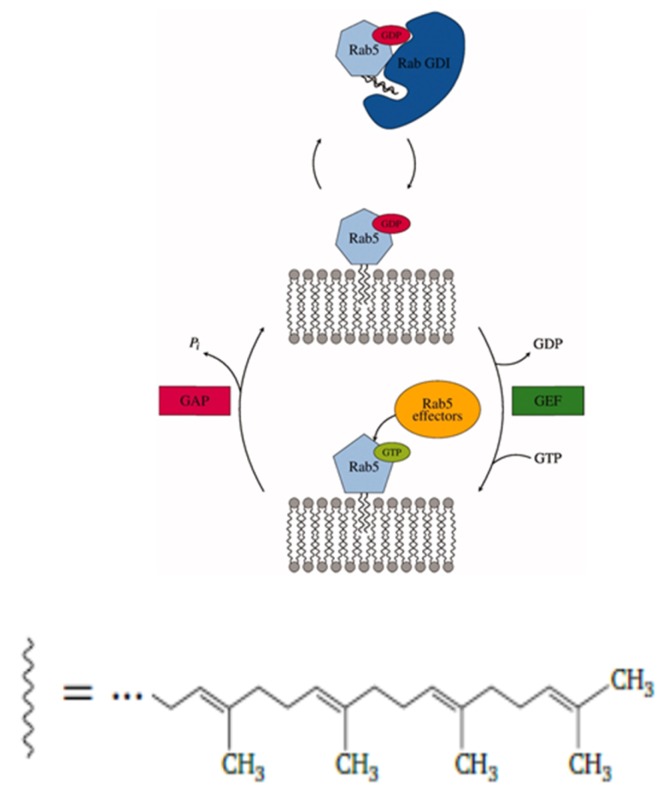
Rab proteins shuttle between inactive, GDP-bound, and active, guanosine triphosphate (GTP)-bound states, which affect their cellular localization. The protein is post-translationally modified and lipidated by a transferase with two 20-carbon lipophilic geranylgeranyl isoprene units. Transport between different vesicular membranes is realized by RabGDI, which exclusively binds Rab(GDP). Guanine nucleotide exchange factors (GEFs) catalyze the exchange of GDP to GTP and thereby the activation of Rab proteins. Rab(GTP) is solely membrane-associated and able to recruit a large number of effector proteins. GTP hydrolysis and recovery of Rab(GDP) is achieved by GTPase activating proteins (GAPs) [5].

Rab5 (Ras gene from rat brain) belongs to the Ras (Rat sarcoma) superfamily of small GTPases (guanosine triphosphatases) and is a key regulator of early endosomal membrane trafficking, fusion, and sorting [1,2,3]. Based on a comparison of sequences, structures, and molecular interaction fields, human Rab proteins could be grouped into six subclusters [4]. As a GTPase, Rab5 cycles between inactive, guanosine diphosphate (GDP)-loaded, and active, guanosine triphosphate (GTP)-loaded states. The activation cycle is controlled by guanine nucleotide exchange factors (GEFs), which replace GDP by GTP and thus activate Rab5, and GTPase activating proteins (GAPs). which accelerate intrinsic GTP hydrolysis and restore the inactive Rab5 state (see Figure 1).

Membrane-bound active Rab5 recruits a large spectrum of effector proteins, which open up an exceptionally complex signaling machinery downstream of Rab5 [6]. Apart from its role in early endosome transport and sorting processes, Rab5 activity is connected to signaling related to the endosome-nuclear pathway [7], receptor tyrosine kinases (RTK) [8], cell cycle, and inflammation [9]. Besides its function in the endo-lysosomal system, Rab5 regulation of phagosome maturation and transport has important implications for pathogen degradation in the immune system [5]. 

Since Rab cellular function is crucial for neurotransmitter release in neurons, Rab5 dysfunctions are associated with several neurodegenerative diseases like Alzheimer’s (AD), Parkinson’s (PD) [10], and Huntington’s (HD) [11]. Rab5 overexpression was shown to attenuate the polyglutamine toxicity, probably due to Rab5 function in early autophagosome formation [12].

**Figure 2:**
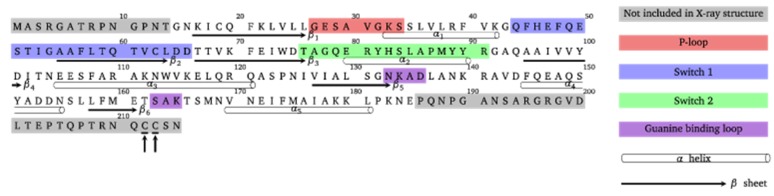
Amino acid sequence of the Rab5 protein and important functional regions. From the 215 residues, only the catalytic G domain structure from Gly15–Glu185 is experimentally resolved. The post-translationally attached geranylgeranyl chains are located at C-terminal residues Cys212 and Cys213 (marked with arrows).

Apart from the G domain, which is conserved among all small GTPases, these proteins exhibit flexible N- and C-terminal regions, which are specific for the individual proteins and involved in membrane localization (see Figure 2). The C-terminal hypervariable region (HVR) encompasses sites of post-translation modifications to associate Rab proteins with membranes. As they are peripheral membrane proteins, Rab GTPases are targeted to specific membrane compartments in order to fulfill their diverse functions in membrane trafficking. In these terminal parts, members from the Ras, Rab, Rho, and Arf subfamilies, contain sequence motifs for post-translational modifications by covalent attachment of hydrophobic chains, which enable membrane anchorage. Membrane attachment is realized via various types of lipidifaction, such as isoprenylation, palmitoylation, or myristoylation, or via an accumulation of positively charged amino acids [13,14]. In the case of the Rab5 GTPases, the C-terminal adjacent cysteine residues Cys212 and Cys213 are both connected to two 20-carbon lipophilic geranylgeranyl isoprene units.

The amino acid sequence of the Rab5 small GTPase with highlighted functional domains is shown in Figure 2. Geranylgeranylation of one or two cysteine residues at the C-terminus of the Rab protein is catalyzed by the Rab geranylgeranyl transferase (RGGT) in concert with the associated Rab escort protein (REP) [15]. First, the heterodimeric RGGT associates with REP and this preformed complex binds unprenylated Rab and transfers the geranylgeranyl moieties from a geranylgeranyl pyrophosphate (GGPP) to the Rab cysteine residues. Pyrophosphate is recycled by the GGPP synthase to form GGPP, which is used in a subsequent transfer step [16]. 

Most Rab proteins undergo double geranylgeranylation to be correctly targeted and inserted into the membrane. Only a few Rab proteins possess a single GG motif responsible for membrane anchoring, e.g., Rab8 and Rab13 [17]. It has been shown that different membrane-binding motifs are highly specific for the protein cellular localization [18]. In addition to the isoprenyl groups, which insert into the bilayer hydrophobic region to anchor the protein, small GTPases like K-Ras4B, Rac-1, Rho-A, or Rap1-A often contain an adjacent cluster of basic amino acid residues to bind negatively charged phospholipid headgroups of specific membranes [19]. On the other hand, N-Ras and H-Ras are farnesylated and exhibit an additional palmitoylation at a C-terminal cysteine residue. When this palmitoylation is missing, both Ras proteins are not correctly targeted to the cytoplasm and are nonfunctional [20,21]. Two-fold GG modification of Rab5 is a prerequisite for protein targeting to the early endosome (EE) where it regulates vesicle fusion and transport [3]. A comparison between wild type Rab5 and different Rab5 mutants, in which the C-terminal double-GG motif was replaced by double-cysteine and mono-cysteine motifs of other Rab proteins, showed that those proteins were correctly post-translationally prenylated [19], but not functional and not delivered to the EE. 

The long and highly flexible *C*-terminus of Rab proteins, the HVR, is structurally not resolved to date and, thus, the membrane-associated protein part and membrane-induced structural effects on the protein structure and orientation are not accessible by experiment. Molecular dynamics (MD) simulations are a valuable tool to investigate protein structures, dynamics, and protein–membrane interactions on an atomistic level [22,23,24]. MD simulations of a lipidated Ras peptide have shown that a single lipid modification is not sufficient to permanently anchor N-Ras to the membrane due to an inadequate hydrophobic binding energy, which causes an equilibrium of adsorption and desorption processes [25]. An exchange of the C-terminal HVR showed that the HVR of Rab1a or Rab2a did not obstruct Rab5 targeting to the early endosomal membrane. Other hybrid proteins with the Rab5 HVR, however, were not delivered and bound to the EE membrane. Thus, general membrane targeting is not exclusively due to the HVR and additional Rab family-specific (RabF) and Rab subfamily-specific (RabSF) sequences were identified [26].

We here investigate the effect of the mono-geranylgeranylation of cysteine Cys212 of Rab5 in its GDP- and GTP-bound states on the protein–membrane association, orientation, and diffusional properties of the membrane and the protein. The results are compared to those of a truncated HVR^206–215^ [27] and the double-GG Rab5 in order to investigate the effects of mono- vs. di-geranylgeranylation and protein anchorage on membrane properties. We demonstrate that mono-geranylgeranylation enables a Rab5 EE membrane interaction and association but the protein orientation at the membrane surface is flexible, less structured, and the diffusion of lipids and protein is significantly enhanced. Thus, a nucleotide-state specific effector protein recognition is not feasible and the mono-GG Rab5 cannot be functional.

## 2. Results

### 2.1. Membrane Properties

Membrane anchoring of the (GG,GG)-HVR of Rab5 affects the local properties of the three different membrane systems. Compared to pure POPC, cholesterol insertion in the other bilayers caused significant increases in global membrane thickness and lipid acyl chain order and a decrease in the area per lipid (the cholesterol condensing effect). The addition of cholesterol increased the overall membrane thickness by inserting between the phospholipid hydrophobic chains in an extended conformation, thus leading to a denser packing and a decrease in area per lipid. The effect can be observed for the truncated HVR^205–216^ and mono- and di-geranylgeranylated Rab5 proteins with almost identical values for the membrane thickness indicating that a) the GG–membrane interactions are very similar for all three systems; and b) protein attachment to the GG-anchor does not affect the membrane thickness (c.f. HVR^206–215^ and double-GG Rab5).

The lipid areas for the HVR^205–216^ and (GG,GG)-Rab5 systems are very identical to within 0.01–0.005 nm^2^, which shows that di-geranylgeranylation has the major influence on lipid condensation independent of the attachment of a protein. For the singly GG-Rab5, however, the area per lipid is larger but the cholesterol condensation effect can also be observed. Obviously, the perturbation of the membrane is less pronounced by a single geranylgeranyl chain only.

The order within the bilayer, or more exactly the acyl chain order, is measured from NMR quadrupolar splitting in experiment [28]. For this purpose one determines the spatial motion of a carbon–hydrogen vector in the lipid acyl chain. The calculation of lipid order parameters was performed using the approach of Douliez [29] for the saturated *sn*-1 palmitoyl chains of POPC, PSM, POPE, and POPS. A medial acyl chain order parameter was determined by averaging over all order parameters of one palmitoyl chain (carbon atoms C2 to C15), see [27]. With increasing cholesterol content, the lipid order significantly increased from 0.17–0.18 in pure POPC to 0.33 in the ternary and 0.31 in the six-component membrane system. This observation reveals that, in particular, membrane thickness and lipid order parameters are not very sensitive probes to discriminate between mono- and di-GG anchored proteins.

### 2.2. Self-Diffusion of Lipid Molecules in the Membrane

The lateral diffusion of lipids can be easily measured and is a versatile probe for comparing the results of MD simulations with experimental data. The movements of individual particles follow Brownian motion, i.e., the motion’s directions are independent from previous directions and random, which means that they can be described by random walk theory. However, since mean square displacement (MSD) leads to averaged diffusion coefficients, it gives inaccurate results for different particle types or particles with different modes of motion. MSD was also shown to give large deviations of the diffusion coefficient D for small ensembles with <100 particles or short trajectories [30]. To circumvent this drawback, the jump distance analysis (JDA) method was used here, which is based on the concept of single-particle tracking, i.e., it tracks the movements of individual particles throughout the simulation and identifies subpopulations by curve fitting [31,32]. This JDA approach was used in the present study to investigate the diffusion of the protein and the GG anchor. 

In pure POPC, the lipid diffusion determined by JDA was 1.36 × 10^−7^ cm^2^·s^−1^ (Table 1), which is in very good agreement with experimental NMR studies [33]. In the cholesterol-enriched ternary and six-component membranes, POPC diffusion was 0.78 × 10^−7^ cm^2^ s^−1^ and 0.47 × 10^−7^ cm^2^ s^−1^, respectively; that is a reduction of approximately 43%. Among the different lipids, POPC diffuses fastest and cholesterol diffusion is slowest. However, the spectrum of diffusion coefficients is in a narrow range from 0.59 × 10^−7^ cm^2^ s^−1^ to 0.74 × 10^−7^ cm^2^ s^−1^ in the six-component membrane. As a consequence of the cholesterol condensing effect, an overall decrease of the lateral lipid diffusion coefficients can be observed. 

### 2.3. Protein Diffusion in Different Membranes

When the Rab5 protein is membrane-associated, its position at the membrane is also dynamic and it diffuses ‘freely’ in the fluid lipid bilayer [34,35].

Table 2 gives the calculated diffusion coefficients of the truncated HVR^206–215^ and mono- and di-geranylgeranylated Rab5. The results clearly show that protein lateral diffusion was slower in complex biomembranes than in artificial model membranes. 

The HVR^206–215^ model diffused fastest in the pure POPC system. When cholesterol was included, we saw a clear slowing down of diffusion when the amount of cholesterol, but also when the overall charge of the membrane, changed. Due to the cholesterol-induced increase in lipid order, the diffusion of the GG chain decreased by almost 50% from 0.78 (in pure POPC) to 0.47 (in the ternary membrane) and 0.43 × 10^− 7^ cm^2^ s^− 1^ (in the six-component charged membrane). The same effect could be seen for the twofold (GG,GG)-Rab protein. 

For mono-GG full-length Rab5, however, the situation was different. MD simulations of 200 ns length in the production phase of mono-GG Rab5(GDP) and Rab5(GTP) in the three different model membranes gave lateral diffusion coefficients of the GG anchor which were 1.7 times (in pure POPC) to 3.5 times (in the six-component membrane) larger compared to the double-GG Rab5 (see Table 3). 

Also, the mono-GG anchor diffusion coefficient was larger in the six-component membrane compared to the ternary mixture. This indicates that characteristic protein–lipid interactions, which are responsible for the decrease of diffusion for (GG,GG)-HVR and (GG,GG)-Rab5, were apparently not present to the same extent between mono-GG Rab5 and the six-component bilayer. 

In the case of full-length (GG,GG)-Rab5, a number of those interactions were formed not only between the negatively charged PI(3)P phospholipid and the HVR, but also between charged lipids and amino acid residues of the protein G domain, thus notably reducing the diffusion in the six-component membrane [36].

### 2.4. Local Membrane Perturbations by Geranlygeranyl Anchors

**Figure 3:**
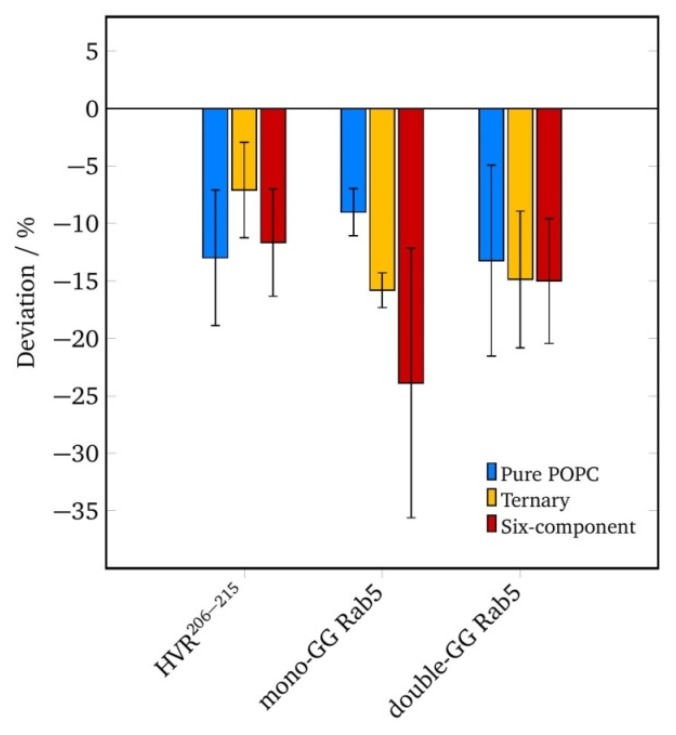
Variation of the average acyl chain order parameter in vicinity to the GG anchor (in a cutoff radius = 0.5 nm) from the global average in the three investigated membrane models for different protein systems.

In order to investigate local perturbations of the membrane structure by geranylgeranyl insertion, the local lipid order parameter was calculated in a radius of 0.5 nm of the GG anchor (see Figure 3). 

The acyl chain order parameter is a measure of the relative positioning of the hydrogen/deuterium atoms of the methylene groups. The acyl chain order parameters were calculated, averaged over all carbon atoms of one chain, and mapped onto the grid. Relative local properties were averaged over the entire 200 ns of simulation and shown as the difference from the global average value.

For the truncated HVR^206–215^, the local lipid order parameters varied by up to 20% in this cutoff radius with the deviations being most prominent in a pure POPC membrane. Independent of the lipid composition, the acyl chain order was reduced in vicinity to the GG anchor compared to the global lipid order parameter in Table 3. The twofold geranylgeranylated Rab5 displayed a local order reduction comparable to that of the HVR^206–215^. 

The GG-Rab5, however, showed a drastic reduction in local lipid order in the ternary and six-component membranes. This increase in lipid disorder was responsible for the increase in lateral diffusion coefficients in the order POPC > three-component > six-component membrane, which facilitated the motion of the protein at the membrane. 

To sum up, all local deviations from the global properties were related to the reduction of lipid order parameters in vicinity of the GG anchor(s) and changes in lateral diffusion coefficients. Thus, one obtains an internally consistent understanding of membrane perturbations and protein diffusion.

### 2.5. Lipid Anchor Insertion into Membrane and Protein–Membrane Distance 

In order to investigate whether the protein–membrane interactions would be different between (GG)- and (GG,GG)-Rab5, the insertion depth of the GG anchor chains into the membranes was investigated. 

**Figure 4:**
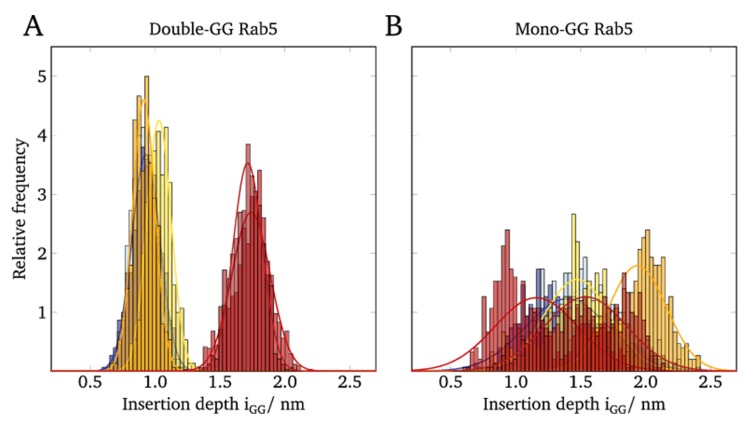
Distribution of the GG anchor insertion depths of (**A**) double-GG Rab5 and (**B**) mono-GG Rab5 in different membranes.

The anchor penetration is calculated as the z-distance between the most remote GG carbon atom and the phosphorous atom of the surrounding phospholipid headgroups [5]. The distributions of the anchor insertion depths of mono-GG Rab5 compared to double-GG Rab5 are shown in Figure 4. 

For the (GG,GG)-Rab5 in the six-component membrane, the insertion depths were normally distributed with average values of 1.74 nm for Rab5(GDP) and 1.71 nm for Rab5(GTP) (see Figure 4A). The insertion depths were expected to hardly differ between the nucleotide states since NMR studies show that the lipid anchor insertion depth adapts to the lipid thickness in order to avoid unfavorable hydrophobic clashes and mismatches [37]. 

Since the insertion depths in pure POPC and the ternary membrane system were significantly less than 0.9–1.1 nm it can be demonstrated that the anchor insertion depth rather depends on the membrane composition than on the activation state of Rab5. 

In contrast to (GG,GG)-Rab5, whose insertion depths clearly depend on the lipid composition and thus, the thickness of the bilayer, mono-GG shows no correlation of the insertion depths with the lipid composition and a rather broad bimodal distribution for both GDP- and GTP-bound states (see Figure 4B). This indicates a very unspecific (GG)-Rab5 membrane interaction and a large variability of Rab insertion depths, independent of the bound nucleotide and the membrane composition.

**Figure 5:**
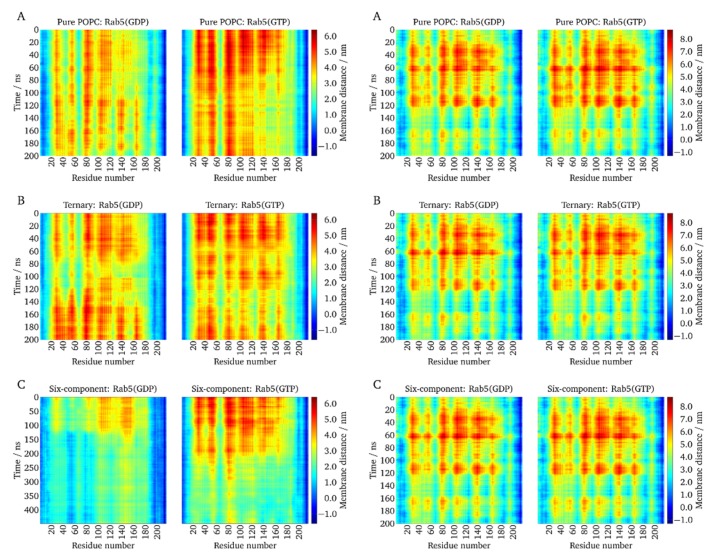
The z-distances between each amino acid residue and the membrane surface were monitored over the molecular dynamics (MD) trajectories. Left: (GG,GG)-Rab5 GDP and GTP; right: (GG)-Rab GDP and GTP.( **A**) in pure POPC, (**B**) the ternary mixture, and (**C**) the six-component membrane.

The dynamics of Rab5 were investigated in more detail. In particular, the distance between the bilayer and the protein was measured as the z-distance between the phospholipid P atoms and the center of mass of each single amino acid. Figure 5 shows the temporal evolution of the Rab5 protein–membrane distances. The absolute protein–membrane distance was at a maximum in the beginning and gradually decreased over the simulated time.

(GG,GG)-Rab5 and (GG)-Rab5 forms in both the GDP- and GTP-bound states were simulated starting from a protein orientation almost perpendicular to the membrane (see above). The absolute protein–membrane distances were largest at the beginning of the simulation and decreased over the simulation time. In the uncharged membranes, i.e., pure POPC and the ternary mixture, (GG,GG)-Rab5(GDP) and Rab5(GTP) performed quite similarly and displayed a similar dynamic behavior and temporal evolution of protein–membrane distances. This is in agreement with the common GG-anchor insertion depth for these two membrane systems.

Protein parts close to residues 50–60, 75–90, and 100–120, and partially around residue 140, showed the largest distances from the membrane surface. These also incorporated residues within or close to the switch regions. 

In contrast, in the six-component membrane, where protein tilting to the bilayer surface was most pronounced, clear nucleotide state-dependent differences could be distinguished [38]. For Rab5(GDP), the distance profile was inverse, that is, residues that were closer to the bilayer surface in Rab5(GTP) were more distant in Rab5(GDP), and vice versa. Besides the Rab5(GDP) termini, the protein domains close to residues 50–60 and 80–90, approximately representing the switch I and switch II regions, were closest to the charged bilayer surface. Apparently, the switch from the perpendicular protein orientation to the tilted orientation occurred after ~120 ns for (GG,GG)-Rab5(GDP) and ~200 ns for (GG,GG)-Rab5(GTP) in the six-component membrane. For (GG,GG)-Rab5(GTP), a switch from the perpendicular orientation to a ‘tilted’ orientation occurred after ~200 ns, which yielded the switch I residues 50–60 and switch II residues 75–92 at a large distance from the membrane, thus making it accessible to effector proteins [5].

For mono-GG Rab5, the G domain hardly approached the bilayer surface in all membrane models. The protein–membrane distance per residue is independent of the nucleotide-bound state (see right hand columns in Figure 5). This indicates that Rab5 membrane anchorage with only one GG chain is sufficient to associate the protein with the bilayer during the MD simulations but not sufficient to induce a G domain bending toward the membrane surface (see below). GDP- and GTP-bound nucleotide states behave similarly and do not allow a state-specific recognition by effector proteins.

### 2.6. Protein Orientation at the Membrane Surface

**Figure 6:**
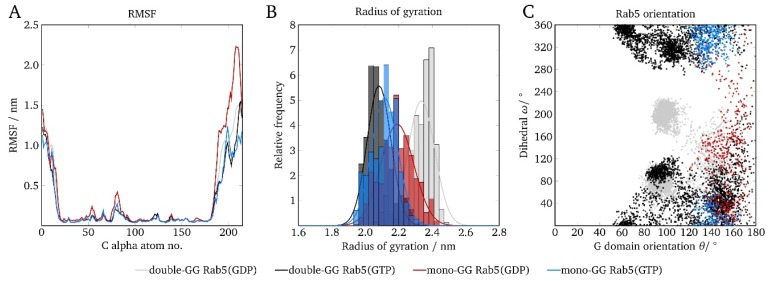
Comparison of mono-GG and double-GG Rab5 structural parameters in the six-component membrane with regard to (**A**) the RMSF, (**B**) the protein radius of gyration, and (**C**) the internal dihedral angle ω as a function of the G domain orientation θ.

The very broad distribution of GG anchor insertion depths (Figure 4) and the absence of GG-Rab5 closely approaching the membrane (Figure 5) are indicators of a large protein flexibility of membrane-associated (GG)-Rab(GDP and GTP). These larger C-terminal structural fluctuations were revealed by the RMSF of mono-GG Rab5(GDP) in the six-component membrane (red line in Figure 6A).

The protein radius of gyration r_gyr_ is described as the distribution of atoms around the protein axis and is calculated as the atomic mass-weighted root mean square distances from the protein center of mass. It therefore characterizes the compactness of a protein structure. The general Rab5 structure compactness measured by the radius of gyration (see Figure 6B) was similar for mono-GG Rab5 in both nucleotide states and close to that of double-GG Rab5(GTP). The larger radius of gyration in double-GG Rab5(GDP) was a consequence of the less ordered switch region structures compared to Rab5(GTP) and the rather extended C-terminus.

In order to describe the protein orientation at the membrane in more detail, the G domain orientation pivot angle θ and the internal dihedral ω can be defined [5]. The dihedral ω as a function of the pivot angle θ is given in Figure 6C.

The (GG,GG) forms of Rab5 show two distinct populations in θ and ω: The internal torsion of the protein regions ω showed two distinct populations for Rab(GDP): one was at ∼80° and the other population was at around 190°. Independent of the membrane system, the G domain orientation takes values between 80° < θ < 160° for (GG)-Rab5(GDP) and 50° < θ < 180° for (GG)-Rab5(GTP). For θ being 90° the G domain adopts a perfectly parallel orientation with respect to the bilayer surface. The internal dihedral ω enables the distinction between an 'endo' and an 'exo' conformation of the switch regions, i.e., the switch regions either facing the bilayer surface or pointing towards the solvent. 

For the double-GG Rab5, these 'exo' conformations were characterized by a strong solvent exposure of the switch regions (ω ≈ 80°). For Rab5(GDP), another large population was found at ω ≈ 190°. These configurations belonged to the switch region’s 'endo' conformations facing the membrane surface. The combination of a G domain orientation almost in parallel to the bilayer surface and the 'endo' conformation of the switch regions caused an efficient screening of the Rab5(GDP) switch regions from solvent in the six-component membrane. The interaction-forming pattern was mainly made up of polar amino acid residues, which form contacts with the lipid head groups. In addition, patches encompassing the positively charged residues Arg39, Lys42, His46, Arg81, His83, and Arg91 were frequently found in close proximity to the membrane surface. This is consistent with previous findings indicating that polar residues play an important role in the reversible adsorption of peripheral membrane proteins [39].

Comparison of the Rab5 orientations in the six-component membrane revealed that mono-GG Rab5 is unlikely to adopt a parallel G domain orientation (θ ≈ 90°) with respect to the bilayer surface (Figure 6C). Furthermore, the switch regions were more frequently found in the ‘exo’ conformation, being fully solvent-accessible in both nucleotide states. Consequently, in contrast to (GG,GG)-Rab5(GDP), mono-GG Rab5(GDP) was at no time found in the tilted conformation with partially solvent-screened switch regions. Thus, there is no discrimination between GG-Rab5 in either GDP- or GTP-bound forms, which would allow a state-specific recruitment of effector proteins. 

**Figure 7:**
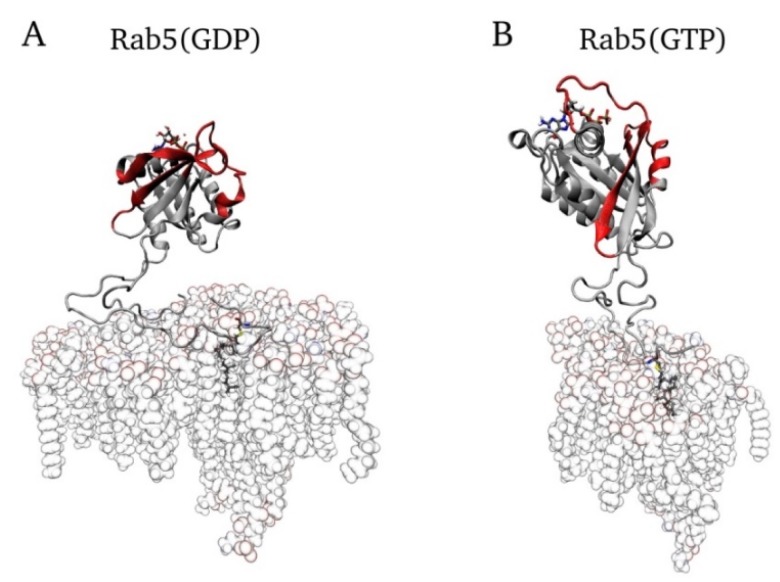
Final conformations of **A**) mono-GG Rab5(GDP) and **B**) mono-GG Rab5(GTP) in the physiological early endosomal six-component membrane.

Figure 7 shows snapshots of the final mono-GG Rab5 conformations in the six-component EE membrane system.

## 3. Discussion

In the present study, the effect of a mono-prenylation of Rab5 in its inactive and active states was investigated and compared with the doubly prenylated Rab5 and the truncated C-terminal domain. Lipidification of only one of the cysteine residues of the HVR was simulated in membranes of different compositions (POCP; POPC/CHOL/PSM; POPC/CHOL/PSM/POPE/POPS/PI3P). The latter six-component membrane mimics the early endosome membrane, i.e. the bilayer that Rab5 is naturally associated with. 

Upon mono-geranylation, the GG-Rab5 G domain hardly approached the bilayer surface in all membrane models. In particular, the significant G domain tilt revealed for double-GG Rab5(GDP) in the physiological six-component membrane was absent for mono-GG Rab5(GDP). This indicates that Rab5 membrane anchorage with only one GG chain is sufficient to associate the protein with the membrane bilayer, but not sufficient to induce a G domain bending toward the membrane surface. The tilting of the Rab5 G domain and the presence of the two distinct orientations for Rab5(GDP) and Rab5(GTP) is essential for a correct protein function, thus only double-GG Rab5 would be able to recruit effector proteins to the membrane surface. In contrast to double-GG Rab5, mono-GG Rab5 reveals only one membrane-binding mode, irrespective of the nucleotide state or bilayer composition. 

Due to the single GG chain anchorage, loose and unspecific protein–membrane interactions impede the formation of long-range electrostatic interactions between the G domain residues and the phospholipids. This is in agreement with the experimental finding that the mono-cysteine motif containing Rab5 was nonfunctional [19]. The number of geranylgeranyl anchor chains significantly affects the anchor membrane insertion depths and the protein diffusion in membranes of different compositions. 

Prenylation of Ras, Rho/Rac, and Rab proteins is essential for proper functioning of the lipidated protein in cellular processes. Until recently, the presence of a di-cysteine prenylation motif at the C-terminus was considered a defining feature of a Rab protein. However, for example, Rab8 and Rab13 possess only a single C-terminal cysteine amino acid. The results explain why GG-Rab5 might be correctly expressed and correctly targeted to the early endosome. Characteristic interactions between membrane and Rab family-specific (RabF) and Rab subfamily-specific (RabSF) sequence patches would be missing and impair its physiological function.

## 4. Computational Details 

### 4.1. Protein Model Generation

Full length Rab5(GDP) and Rab5(GTP) structures were taken from ref. [5] and are based on PDB 1TU4 and 1R2G, respectively. They were shown to give stable MD trajectories for the protein in solution and when bound to the membrane [5,36].

### 4.2. Molecular Dynamics- Computational Details

All-atom MD simulations were performed with NAMD2.9 [40] and the full-atomistic CHARMM36 force fields for proteins and lipids [41,42,43,44].

The parameters for the nucleotides GDP and GTP were adapted from the established parameter set for nucleic acids [45] and combined with phosphate group parameters from ADP/ATP [46]. 

Solvent interactions were modelled using the explicit TIP3P water model [47]. In preparation of the MD production runs, the membrane–protein–water system was energy minimized, heated to 310 K, and equilibrated for 50 ns. The production runs were performed in an ensemble of a constant number of particles, pressure, and temperature, controlled by Langevin dynamics [48]. Periodic boundary conditions were applied and the particle mesh Ewald (PME) [49] method was used to calculate electrostatic interactions. Positions, velocities, and forces were calculated every 2 fs. The Cα atom root mean square fluctuation (RMSF) was computed after superposing the G domain coordinates onto the first frame structure by using the VMD “measure fit” and “measure rmsf” commands.

### 4.3. Parameterization of Geranylgeranyl 

The geranlygeranylated cysteine amino acid (GG-Cys) force field parameters were adapted from the published CHARMM36 topologies for proteins [41] and lipids [42] and the torsional angles probability distributions and energy barriers of the corresponding dihedral angles in the MD trajectories were calibrated against quantum mechanical DFT calculations (see reference [27] for details). The dihedral angles, which corresponded to the energy minima of the quantum chemically calculated torsional profiles were most frequently visited in the MD simulations. This demonstrates the suitability of the GG-Cys force field parameters to accurately reproduce quantum mechanical results.

### 4.4. Composition of Different Membranes

In the present study, three different symmetric model membranes of increasing complexity were used [36]. A neutral pure palmitoyl-oleoyl-phosphatidylcholine (POPC) bilayer was built with the VMD Membrane Plugin [50]. A ternary mixture of POPC, cholesterol, and palmitoyl-sphingomyelin (PSM) in a ratio 2:2:1 served as an example for a simple plasma membrane model often used in experiments with the ability to form liquid-ordered (lo) domains [51]. The six-component model membrane features an overall negative charge due to the presence of palmitoyl-oleoyl-phosphatidylserine (POPS) and phosphatidylinositol 3-phosphate (PI(3)P). PI(3)P is an important signaling lipid primarily enriched in the EE membrane where it serves as a recognition signal that recruits cytoplasmic effector proteins to promote membrane deformation, tethering, membrane fusion, etc. The six-component membrane was therefore used as a complex model system for the EE membrane. The exact lipid compositions of the model bilayers are given in ref [27]. The model membranes were solvated, ionized, energy minimized, and heated to a temperature of 310 K. First, equilibration was performed for 2 ns in a canonical (NVT) ensemble with all atomic coordinates kept fixed, except for the lipid tails, in order to introduce sufficient disorder in the tails region. In a second equilibration step, the fully unconstrained membrane system was simulated for another 50 ns in an isothermal–isobaric (NPT) ensemble. After these steps, protein and membrane systems were combined [27]. Details of the simulation box dimensions and number of lipid molecules and number of solvent molecules, sodium, and chloride ions is given in the Supplementary Information Table S1.

### 4.5. Starting Configuration

In all protein–membrane simulations, the bilayer was placed in the x, y-plane and the protein was localized in +z direction above the membrane surface. Membrane anchoring occurred via the *C*-terminal GG chains, which were inserted by superposing the prenyl groups with the lipid tails of one POPC molecule. Afterwards, the corresponding POPC lipid was deleted. In the case of full-length protein, one Rab5 entity was membrane-anchored by one GG-Cys residue and the G domain of Rab5 was translated to a position perpendicular to the membrane. 

**Figure 8:**
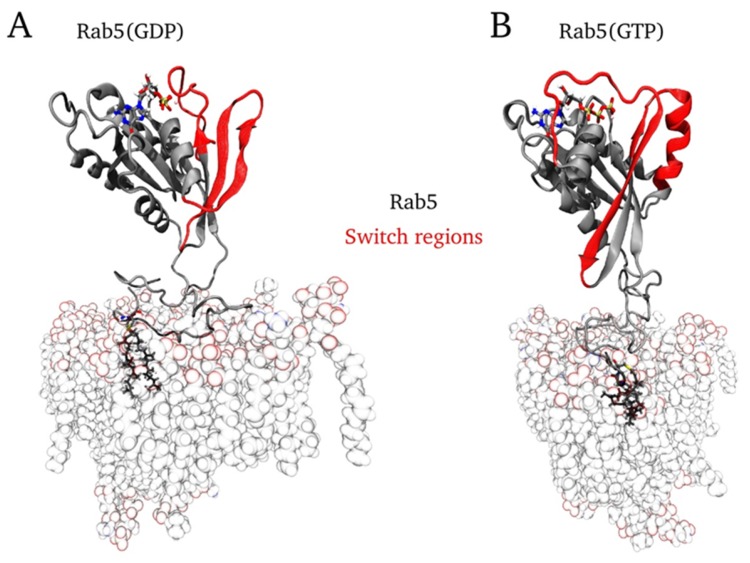
Initial conformations of full-length membrane-bound (**A**) Rab5(GDP) and (**B**) Rab5(GTP) (here in the six-component membrane), which was identical for either one- or two-fold geranylgeranylated HVR.

All membrane-bound Rab5(GDP) and Rab5(GTP) MD simulations started from a protein orientation perpendicular to the bilayer surface (see Figure 8).

Mono- and di-GG Rab5(GDP) and Rab5(GTP) were anchored to each membrane model and subjected to 200 ns production MD simulations after 50 ns of equilibration. The equilibrated six copy HVR^206–215^ simulation in different membrane models [27] was used for comparison. For the complex six-component membrane, double-GG-Rab(GDP)/(GTP) 500 ns trajectories from [5,36] were also used for comparison. Figure S1 in the Supplementary Information gives results of the additionally performed double-GG-Rab(GDP)(/GTP) simulations: the protein RMSD of the double-GG Rab5(GDP and GTP) Cα atoms from the initial configuration over the complete MD simulation in pure POPC, the ternary mixture, and the six-component membrane. Figure S2 shows the protein RMSD and the temporal evolution of the area per lipid molecule, bilayer thickness for the mono-geranylgeranylated Rab5 in GTP-and GDP-bound states in membranes of different composition. Averages with standard deviations are given in Table 1.

### 4.6. Geometrical Analysis

The insertion depth of the GG chains within the bilayer was calculated as the z-distance between the neighboring lipid P atoms and the most deeply inserted carbon atom of the GG chains. The distance between the bilayer and the protein was measured as the z-distance between the phospholipid P atoms and the center of mass of the single amino acids. The calculation of acyl chain order parameters was performed using the approach of Douliez et al. [52] for the saturated palmitoyl chains of POPC, PSM, POPE, and POPS at the glycerol sn-1 position. For PC, PE, PS, and PSM, the head group orientation is defined as the angle between the bilayer normal, n, and the direction vector between the phosphorous and nitrogen atoms (PN vector), see ref [27] for more details. Two observables describe the Rab5 orientation at the membrane: the pivot angle θ reflecting the G domain orientation, → G, and the dihedral φ depicting the torsion between membrane, G domain and the switch regions (for more details see [5]).

## Figures and Tables

**Table 1:** Lateral lipid diffusion coefficients in 10^−7^ cm^2^·s^−1^ in pure POPC, the ternary, and six-component membranes.

Membrane	System	POPC	Chol	PSM	POPE	POPS	PI(3)P
Pure POC	HVR^206–215^	1.36					
	GG-Rab5	1.63 ± 0.08					
	(GG,GG)-Rab5	1.44 ± 0.03					
Ternary Membrane	HVR^206–215^	0.78	0.59	0.60			
	GG-Rab5	0.83 ± 0.03	0.65 ± 0.05	0.72 ± 0.04			
	(GG,GG)-Rab5	0.75 ± 0.02	0.55 ± 0.02	0.64 ± 0.02			
Six-Component Membrane	HVR^206–215^	0.74	0.59	0.62	0.68	0.65	0.62
	GG-Rab5	0.79 ± 0.01	0.62 ± 0.02	0.66 ± 0.02	0.73 ± 0.01	0.70 ± 0.03	0.67 ± 0.03
	(GG,GG)-Rab5	0.74 ± 0.01	0.58 ± 0.01	0.61 ± 0.01	0.67 ± 0.01	0.64 ± 0.01	0.60 ± 0.01

**Table 2:** Lateral diffusion coefficients of the geranylgeranyl (GG) anchor in different membranes.

Diffusion Coefficient/ 10^−7^ cm^2^ s^−1^	HVR^206–215^	Mono-GG Rab5	Double-GG Rab5
Pure POPC	0.78 ± 0.13	1.59 ± 0.33	0.93 ± 0.09
Ternary Membrane	0.47 ± 0.10	1.31 ± 0.30	0.51 ± 0.06
Six-component Membrane	0.43 ± 0.08	1.49 ± 0.08	0.43 ± 0.05

**Table 3:** Global thickness, lipid area, and average lipid order parameter of the three model membranes when simulated with different peptide/protein systems.

Parameter	System	Pure POPC	Ternary Membrane	Six-Component Membrane
Membrane thickness/ nm	HVR^206–215,a^	3.87	4.60	4.54
	Mono-GG Rab5^b^	3.88 ± 0.02	4.61 ± 0.01	4.57 ± 0.01
	Double-GG Rab5^c^	3.88 ± 0.03	4.61 ± 0.02	4.56 ± 0.02
Lipid area/ nm^2^	HVR^206–215,a^	0.662	0.437	0.466
	Mono-GG Rab5^b^	0.854 ± 0.006	0.534 ± 0.004	0.518 ± 0.005
	Double-GG Rab5^c^	0.652 ± 0.044	0.432 ± 0.002	0.460 ± 0.002
Lipid order parameter	HVR ^206–215,a^	0.176	0.327	0.305
	Mono-GG Rab5^b^	0.174 ± 0.001	0.326 ± 0.002	0.309 ± 0.001
	Double-GG Rab5^c^	0.176 ± 0.001	0.327 ± 0.001	0.309 ± 0.001

^a^ One trajectory with 6 copies of HVR [27]; ^b^ average and standard deviations of both nucleotide states; ^c^ Average and standard deviations for triplicate simulations of both nucleotide-bound states.

## Supplementary Materials

Supplementary material (Tables and Figures) can be found at https://www.mdpi.com/1422-0067/20/19/4773/s1. Starting structures (pdb) and psf files to reproduce MD simulations of mono-geranylgeranylated Rab5(GDP)/(GTP) in three different membrane systems can be found at http://doi.org/10.5281/zenodo.3406123.
