# Supplementary material for: Structure and Dynamics of Mono- vs. Doubly Lipidated Rab5 in Membranes
Source: Int J Mol Sci. 2019 Sep 26;20(19):4773. doi: 10.3390/ijms20194773 (PMC6801778; doi:10.3390/ijms20194773)
Supplement: Supplementary file 1 [file ijms-20-04773-s001.pdf]

International Journal of *Molecular Sciences*

MDPI

*Supplementary Information*

# Structure and Dynamics of Mono- *vs.* Doubly-Lipidated Rab5 in Membranes

**Eileen Münzberg[1] and Matthias Stein[1,*]**

[1] Max Planck Institute for Dynamics of Complex Technical Systems, Molecular Simulations and Design Group, Sandtorstrasse 1, 39106 Magdeburg, Germany.

* Correspondence: matthias.stein@mpi-magdeburg.mpg.de

**Table S1**. Dimension of simulations boxes and simulated systems

| Membrane system | Component | Number of lipids (ratio) | Lateral (x,y) dimensions / nm |
|---|---|---|---|
| Pure POPC | POPC | 2 x 273 (100%) | 15.7 x 14.9 |
| Ternary | POPC | 2 x 166 (40%) | 14.8 x 14.4 |
| | CHOL | 2 x 166 (40%) | |
| | PSM | 2 x 83 (20%) | |
| Six-component | POPC | 2 x 90 (17.8%) | 16.9 x 16.6 |
| | CHOL | 2 x 150 (29.7%) | |
| | PSM | 2 x 50 (9.9%) | |
| | POPE | 2 x 135 (26.7%) | |
| | POPS | 2 x 55 (10.9%) | |
| | PI(3)P | 2 x 25 (5.0%) | |

Simulation z-direction box dimensions
12.8 nm HVR,
16.4 nm GG-Rab5(GDP), 16.4 nm GG-Rab5(GTP),
16.9 nm G-Rab5(GDP), 17.0 nm G-Rab5(GTP).

The exact number of water molecules and ions were in the charged six-component membrane
HVR$^{206-215}$ 65586 water/186 chloride/440 sodium
GG-Rab5(GDP) 94096 water/266 chloride/524 sodium
GG-Rab5(GTP) 94426 water/267 chloride/526 sodium
G-Rab5(GDP) 94805 water/268 chloride/526 sodium
G-Rab5(GTP) 97284 water/275 chloride/534 sodium

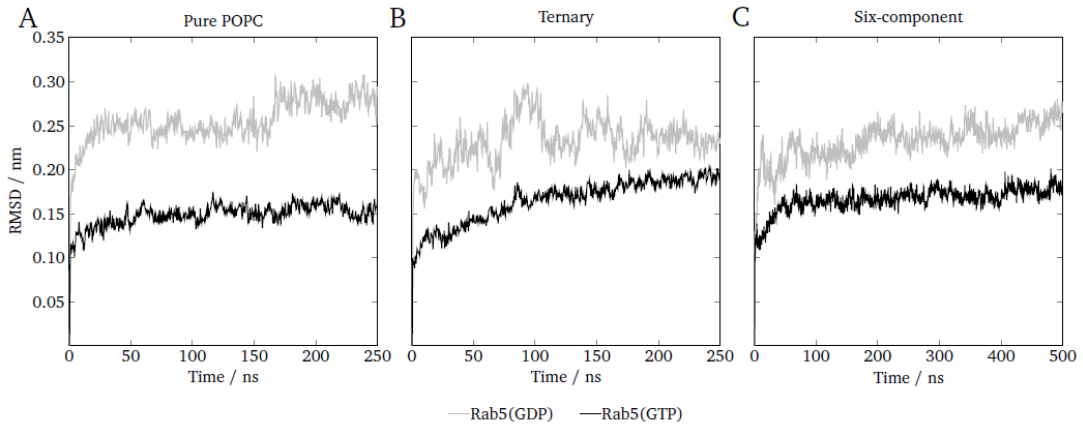


**Figure S1**. Top: RMSD of the double-GG Rab5 Cα atoms from the initial configuration over the complete MD simulation in A) pure POPC, B) the ternary mixture, and C) in the six-component membrane. Data are averaged over three independent simulations for Rab5(GDP) or Rab5(GTP) in each membrane system.

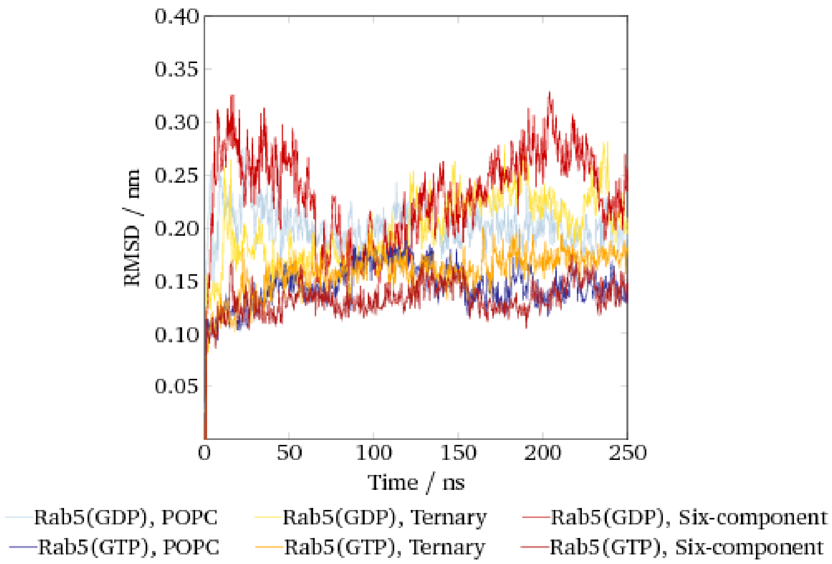


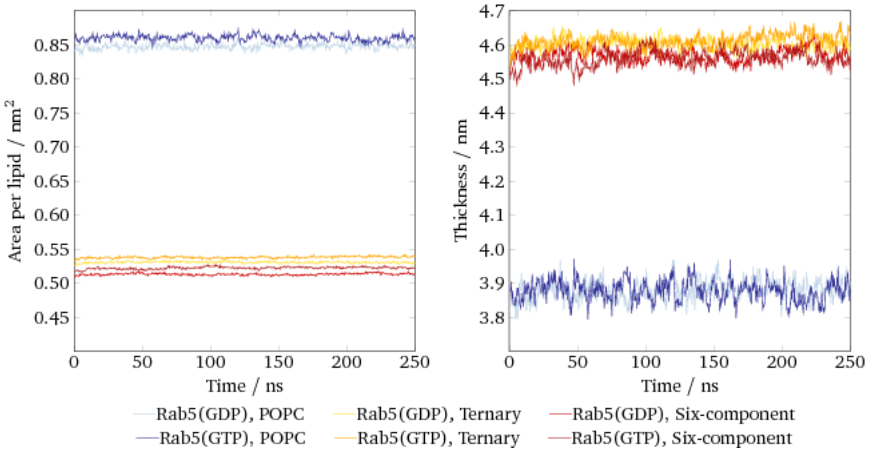


**Figure S2**. RMSD (top), area per lipid molecule and bilayer thickness (bottom) for mono-geranylgeranylated Rab5 in GTP-and GDP-bound states in membranes of different composition (POPC, ternary and six-component membranes; see text for details).

© 2019 by the authors. Submitted for possible open access publication under the terms and conditions of the Creative Commons Attribution (CC BY) license (http://creativecommons.org/licenses/by/4.0/).
